# Well Done, Montana! Well Done, Knowles!

**Published:** 1901-03

**Authors:** 


### EDITORIALS.

### WELL DONE, MONTANA! WELL DONE, KNOWLES!

SINGLE-HANDED and alone our esteemed colleague, State Veterinarian Knowles, in the face of the most serious opposition in the Senate and House, has passed one of the most comprehensive meat-inspection and milk-inspection bills ever engrafted upon a State by any legislative body. We print it in full in this number and it speaks for itself. It is strong, clear in its purposes and intent, broad in its aims and desires, and when honestly and efficiently enforced will give to that State blessings she has never before tasted or enjoyed. Dr. Knowles deserves the greatest credit for his efforts, and the friends of the veterinary profession among the list of medical practitioners who aided in its achievement are to be sincerely thanked, while the support given by the press is fully appreciated, and it is warmly commended for its zeal and interest in the public welfare, where personal interests were sure to be involved and imperiled.

We congratulate Montana on her splendid law and for her high planes of advancement along sanitary lines, and the more enlightened East may well spend a period in sackcloth and ashes as they mourn over defeated or vicious legislation that is supposed to stand for such conditions as can be remedied only by wise police sanitary regulations. The American Veterinary Medical Association might well go West to Montana in 1902 to bask in the sunshine of successful legislation and receive the stimulus and encouragement for future success in other States through the efforts of such workers as Knowles, of Montana.

